# Round the Hospitals

**Published:** 1916-08-05

**Authors:** 


### ROUND THE HOSPITALS.

Hospitals, and especially the larger hospitals with training schools, are believed by the public to be the centre of knowledge in regard to the prevention of disease and the inculcation of personal hygiene. Lord Lister's teaching, and the modern experience and practice based upon it, have enforced with tenfold power the importance of aseptic treatment and aseptic methods. A correspondent who moves about much in London has been shocked of late by the appearance of trained nurses, in full indoor uniform with the addition of a bonnet, in public vehicles, in the street, and among the crowds of people attending places of amusement, moving about in shops and outside. In and about Wandsworth, Army nurses in numbers may be met wearing indoor uniforms. We are informed that the indoor uniforms of the leading hospitals are seldom or never seen in such places, but that of late the London uniform is to be met with nearly everywhere in the circumstances named. The rank of the nurses who so act as gatherers up of impurities of all kinds from the streets, public vehicles, and crowds, which they carry with them into their wards or the sick-rooms of their patients, can be known from the silver and other badges worn by them. It is not easy to exercise complete supervision over a staff of women who must be counted in hundreds, and we call attention to the dangers and abuses in question in the hope of helping the matrons and authorities to save the sick from the risks which may arise in consequence. Guy's Hospital nurses are not allowed to appear outside the hospital in their washing dresses. In the case of Queen's nurses they are forbidden to wear their aprons anywhere except in the patient's house. How many hospital authorities permit their nurses to wear their indoor uniform, aprons, and badges in the public streets?

---

We have received a report on the condition of the sleeping quarters provided at the King George Hospital—a hospital, remember, which bears His Majesty's name—which makes them out to be so bad as to be almost unbelievable. There seems to be an utter disregard for all hygienic considerations, and members of the staff, quite irrespective of their rank, are, we are informed, huddled together in small, ordinary rooms, where a continuous interchange of air is impossible, and in numbers so great as to produce a state of overcrowding of the most flagrant character. King George Hospital has never really settled down into a state of high efficiency, and now we have this revelation of a condition of affairs which would disgrace the most disreputable institution in the Poor-Law system. It calls for the interference of those in authority. If King George, who has a practical knowledge of hospitals, could take an early opportunity to call at the King George Hospital and go straight to the accommodation for the sisters and nurses, he might learn something and render practical service. The effects of such a visit could not fail to have influences for good throughout a system which has permitted these evils to continue, and may do so until the war is over, unless a stronger hand is introduced forthwith to secure justice for the staff and to display some regard to the high standard of efficiency which we have the right to expect to find in the King George Hospital.

---

The mischief of it is that our report states that members of the staff are breaking down, as well they may do. Fancy the state of the atmosphere throughout the night at this season in these crowded quarters for nurses in Stamford Street, Blackfriars! Remember, too, the effect of keeping a nursing staff under such conditions to attend to crowded wards and struggle on with their daily duties until they break down and are passed on under the Government compensation scheme to be quietly disposed of. Could ineptitude all round attain a higher standard of failure and disgraceful results than that which is declared to prevail at the King George Hospital?

---

Perhaps it is the hot weather, but whatever be the reason, our office is full of complaints this week. There seems to be good ground for those made by district nurses and members of the staff of Poor-Law infirmaries accompanying patients, and the patients themselves, in regard to the reception met with in the ophthalmic and other departments of some of the hospitals. The same complaint applies to visitors to patients and friends of patients in the wards. The grievance crystallises down to rough and rude treatment, a lack of consideration, and a sad absence of courtesy all round. It is said that many hospitals are acquiring an unenviable reputation for discourtesy in these respects, but that the London Hospital stands out as a haven for friends and patients owing to the enforcement by the authorities there of a wiser and better system. Great care has been taken, we understand, to test the manners and methods of the staff at the London, with results which have tended to give it this better reputation. It would be a good thing if other chairmen of hospitals would make it their business to test the manners and methods pursued towards patients and visitors in the out-patient dispensaries departments, and at the entrance on visiting days at their institution. Every matron can help her hospital by devoting time and thought to this matter, with a view to secure the maximum of courtesy, and the minimum of friction where patients and their friends are concerned.

---

At King's College Hospital the practical matters in which the probationers are expected to make themselves proficient have been carefully thought out, and represent the high-water mark of modern training. (1) Dressing of blisters, burns, sores, wounds, applying fomentations, poultices, and minor dressings, the application of leeches, the administration of subcutaneous injections. A thorough knowledge of the various dressings, lotions, surgical and medical appliances in use. (2) The administration of enemata for men and women, and the use of the catheter for women; the management of trusses and appliances in uterine complaints. (3) The management of helpless patients. (4) Bandaging, making bandages, padding splints, temperature-taking, charting, etc. (5) Bed-making and removing sheets whilst patient is in bed. (6) Attending upon operations and learning the management of the operating-theatres and all pertaining to them. (7) Cookery for the sick and convalescent. (8) The ventilation of wards, use of disinfectants, the careful treatment of the nurse's own hands. (9) The careful observation of the following particulars respecting patients:—Secretions, expectoration, pulse, skin, appetite, delirium, breathing, sleep, etc.; effect of diets, stimulants, and medicine; giving a concise and intelligent report of the patient's condition. (10) The management of convalescents.

For Matrons' and Sisters' Appointments and Military Needs see p. vi; see also Editor's Letter-Box, p. 437.

